# JAK out of the Box; The Rationale behind Janus Kinase Inhibitors in the COVID-19 setting, and their potential in obese and diabetic populations

**DOI:** 10.1097/XCE.0000000000000237

**Published:** 2020-10-15

**Authors:** Rahma Menshawey, Esraa Menshawey, Ayman H.K. Alserr, Antoine Fakhry Abdelmassih

**Affiliations:** a Research Department, Students’ and Interns’ Research Program (Research Accessibility Team), Cairo University Kasr al Ainy Faculty of Medicine; b Department of Vascular Surgery; c Pediatric Cardiology Unit, Pediatrics’ Department, Faculty of Medicine, Cairo University Hospital, Cairo, Egypt

**Keywords:** baricitnib, COVID-19, diabetes, Janus kinase-signal transducer and activator of transcription inhibitor, obesity, ruxolitinib

## Abstract

The adaptive use of Janus kinase (JAK)-inhibitors has been suggested by rheumatology experts in the management of COVID-19. We recount the rationale behind their use in this setting, and the current evidence for and against their use in this review. JAK-inhibitors role in COVID-19 infection appears to be multifaceted, including preventing viral endocytosis and dampening the effect of excessive chemokines. This drug class may be able to achieve these effects at already preapproved dosages. Concerns arise regarding reactivation of latent viral infections and the feasibility of their use in those with severe disease. Most interestingly, JAK-Inhibitors may also have an additional advantage for diabetic and obese populations, where the dysregulation of JAK-signal transducer and activator of transcription pathway may be responsible for their increased risk of poor outcomes. Targeting this pathway may provide a therapeutic advantage for these patient groups.

## Introduction

The urgency caused by the pandemic of coronavirus disease 2019 (COVID-19) has resulted in the frantic search and repurposing of many medications in the quest to treat it. This includes a wide array of antiparasitic, antiviral, antibiotic and immunological mediations [1–6].

COVID-19 is characterized by a state of pulmonary hyper-inflammation and cytokine storm [7], the suggested culprit of which is interleukin-6 (IL-6) as well as other cytokines [8,9]. The challenge in treating COVID-19 lies in finding the fine line where the immune system response is modulated with enough precision so that the infection is dealt with, while at the same time avoiding the detriments of an aggravated immune response. In light of this, a paradigm shift has occurred and is reshaping how we target inflammation in the setting of infection: to achieve the right response, in the right way and the right amount.

Focus on the inflammatory dysregulation, which is the driving force behind COVID-19 morbidity and mortality, has opened the grounds for drugs such as immunologicals [8].

Of particular interest are Janus kinase-signal transducer and activator of transcription (JAK-STAT) inhibitors and their potential in treating COVID-19 patients, as initially suggested by Richardson *et al*. [10]. The JAK-STAT pathway plays a critical role in coordinating the immune response.

Furthermore, JAK-STAT pathway dysregulation is noted in obese and diabetic populations. Interestingly, among those patient groups, there exists a higher risk for more severe disease and poor outcomes in COVID-19 infection. We outline here the rationale behind the use of JAK-STAT inhibitors in the setting of COVID-19 infection, including their potential for use in diabetic and obese subgroups and provide suggestions for healthcare practitioners.

## The rationale

### Inflammation and viral endocytosis

The JAK-STAT pathway involves a family of proteins that are involved in a myriad of cellular processes, including cell division and immunity [11]. The importance of this pathway in defense against infection is evidenced by the fact that many organisms have adapted methods [12] that target JAK-STAT proteins for their survival. Additionally, the occurrence of some immunodeficiencies is the result of mutations in JAK interactions [13].

**Fig. 1:**
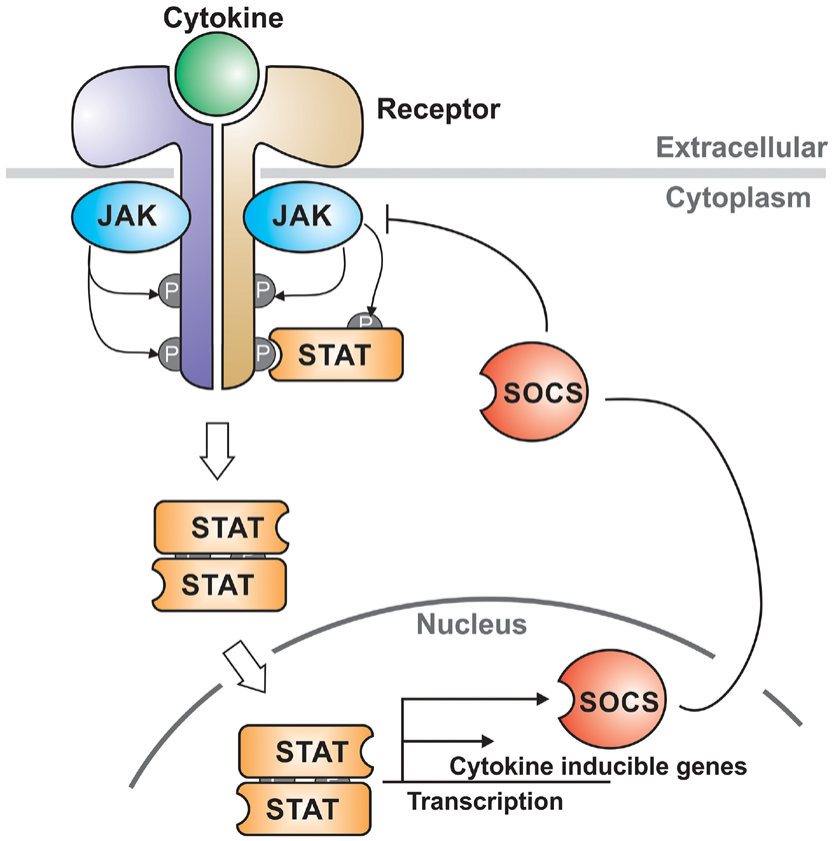
The JAK-STAT pathway. Cytokine binds to the receptor which activates JAK-STAT. STAT homodimers are translocated into the nucleus, where they go on to upregulate the transcription of cytokine responsive genes. Reused with permission (lisence number: 4861540664915). JAK-STAT, Janus kinase-signal transducer and activator of transcription; SOCS, suppressor of cytokine signalling.

In the simplest terms, activation of this pathway leads to the promotion of several inflammatory products [14]. Upon binding of a chemokine to the JAK-receptor, a cascade of reactions is triggered [15], whereby their transcription is greatly increased (see Fig. 1). In the setting of COVID-19, the overproduction of these cytokines, especially IL-6, is responsible for the event of a cytokine storm. For this reason, immunologicals such as JAK inhibitors are being repurposed in an attempt to dampen this immune response.

**Fig. 2:**
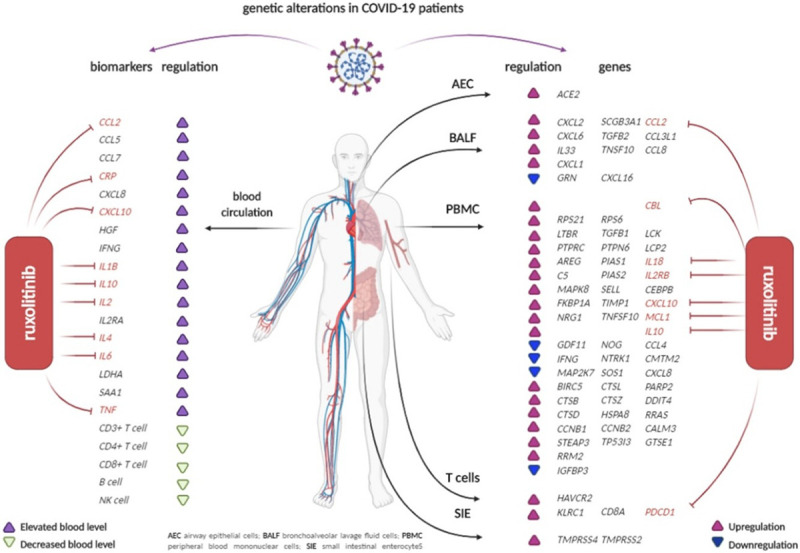
Genetic alterations seen in COVID-19. The JAK inhibitor ruxolitinib appears to target the majority of these alterations. Image reused with permission (license number: 4861521389447). COVID-19, coronavirus disease; JAK, Janus kinase.

JAK inhibitors have also been shown to target the specific genetic alterations observed in the COVID setting, including c-reactive protein, IL-2, IL2RB, IL6, TNF and others [16] (see Fig. 2). They also affect the endocytosis of the virus by means of blocking G-associated kinase and adaptor associated kinase 1 [17]. Artificial intelligence algorithms have pinpointed baricitinib for its affinity in this role; conveniently, it does so at already approved therapeutic dosages. Upadacitinib has been found to be the greatest at reducing levels of IL-6, via inhibition of STAT-3 [18].

### The ACE2 and angiotensin II connection

A connection exists between JAK-STATs and the trans-membrane receptor ACE2 which is the receptor by which severe acute respiratory syndrome coronavirus 2 (SARS-Cov-2) enters body cells [19]. Upon viral entry, ACE2 becomes internalized [20]. The cytokines produced via the JAK pathway have been found to internalize ACE2 receptors, as well. Initially, it was thought that, in studies of the 2002 SARS outbreak [21], these cytokines may decrease susceptibility to infection by decreasing the availability of ACE2 receptors. However, in an already infected person, the loss of the ACE2 protective effects on vascular biology became a matter of concern. This is especially relevant in COVID, where the culprit of symptoms is owed to an inflammatory response and not due to viral load [22].

Interestingly, spike protein and ACE2 interactions activate a disintegrin metalloprotease which results in the total shedding of the ACE2 receptor from the cell surface. This results in the increase in local production and effect of angiotensin II (AII) and hyaluronan; both of which are implicated in the development of acute respiratory distress syndrome [23–26]. Production of AII itself is induced by the activation of JAK signaling [27].

**Fig. 3:**
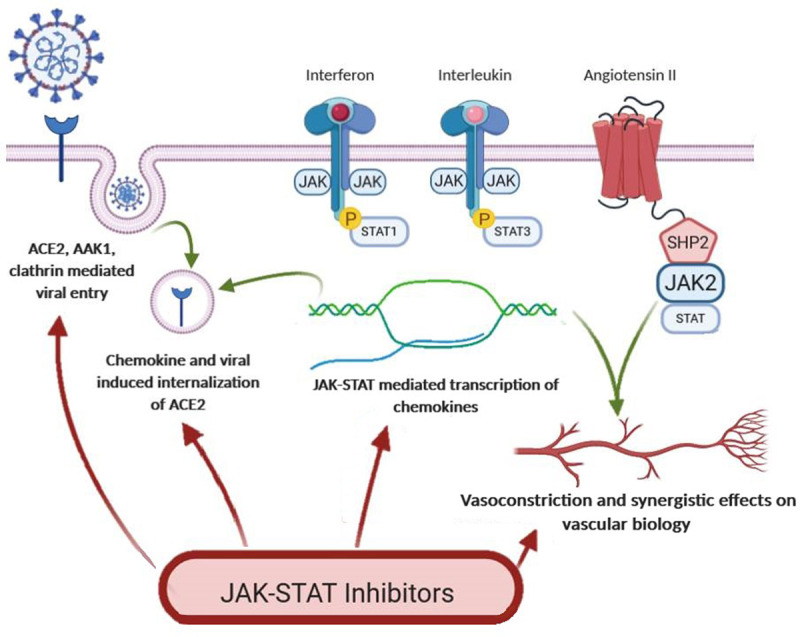
Effect of JAK-STAT inhibition is multifaceted. It may inhibit viral endocytosis, as well as decrease the transcription of inflammatory cytokines, the presence of which is highly implicated in morbidity and mortality in COVID-19, as well as dampen the effect of these chemokines and that of angiotensin II, on vascular biology. JAK-STAT, Janus kinase-signal transducer and activator of transcription.

There are two polarizing hypotheses regarding the inhibition of renin angiotensin system (RAS): (1) inhibition should prove harmful in that ACE2 receptors are increased and available for viral binding, or that (2) inhibition should prove protective by inhibiting the inflammatory/fibrotic effects of AII [28,29]. The decisive factor remains whether or not RAS blockade should increase ACE2 in humans, and in a review of the literatures, it was found that Angiotensin Receptor Blockers/Angiotensin Converting Enzyme Inhibitors did not [30–34]. These findings shift the hypothesis in favor of RAS blockade-related anti-inflammatory effects, as well as dispel fears of medication use in hypertensive patients in the setting of COVID, where they have not been associated with a higher risk of COVID-19 severity or mortality [35–38]. Additionally, the chemokines produced through the JAK pathway play a synergistic role with AII on its effects on vascular biology [39,40] (see figure 3). These effects would be irresistibly blocked with the use of JAK-STAT inhibitors.

## JAK-STAT in Obese and Diabetic Populations

### A dysregulated pathway: a common pathogenesis

The JAK-STAT signaling pathway is dysregulated in obese and diabetic patients and is implicated in disease progression.

In the brain, leptin signaling involves the JAK2 receptor and downstream signaling by STAT3. This causes increased transcription of pro-opiomelanocortin and decreased transcription of agouti related peptide and neuropeptide Y, to the effect of maintaining the anorectic response to leptin. JAK signaling also induces expression of a negative regulator of leptin expression, suppressor of cytokine signalling 3 (SOC3) [41]. In lean persons, leptin induces satiety and energy expenditure, whereas, in the obese person, there is leptin resistance despite high leptin levels, which goes on to suppress the secretion of insulin from B islet cells of the pancreas and promotes hypertension.

**Fig. 4:**
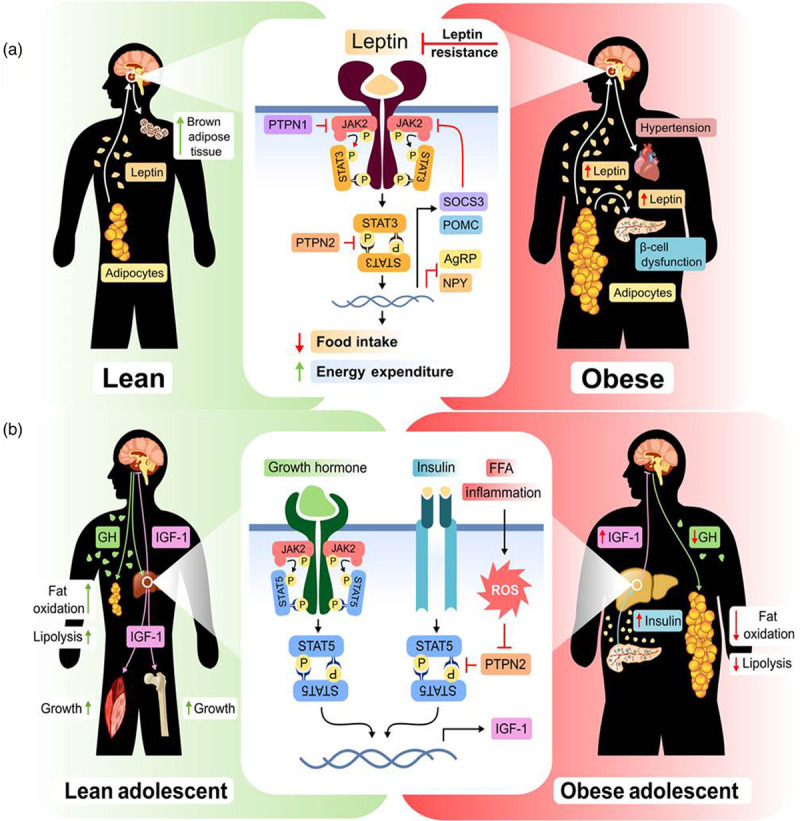
JAK-STAT signaling pathway in obesity. (a) Leptin binds to its receptor and induces JAK-STAT signaling pathway. In the lean person, leptin causes satiety and energy expenditure. In the obese person, there is leptin resistance in spite of high levels of leptin. This hyperleptinemia is further implicated in the inhibition of insulin release from the B islet cells in the pancreas. Aberrant JAK-STAT signaling is implicated in the development of obesity. (b) JAK-STAT signaling is also involved in the growth hormone/insulin-like growth factor-1 axis. Free fatty acid and inflammatory signaling are also implicated in the activation of JAK pathway. All of which is the end result of increased adipose tissue accumulation. Image reused with permission (license number: 4862101271475). JAK-STAT, Janus kinase-signal transducer and activator of transcription.

Mutations in JAK-STAT signaling have resulted in the development of obesity (see Fig. 4) [42]. Hepatic steatosis is, in part, regulated by JAK-STAT signaling – in particular, through its effect on the insulin-like growth factor-1 (IGF-1)/growth hormone axis [43]. STAT1 signaling has been shown to increase fatty acid uptake and steatosis. STAT5, triggered by obesity-mediated hyperinsulinemia, was shown to increase IGF-1, which exacerbates steatosis and also explains the enhanced growth rates observed in obese children and adolescents [44,45]. Knockouts in STAT pathways showed adipocyte hypertrophy and an increase in weight.

In diabetes, JAK-STAT signaling contributes to the disease process of both forms. In type 1 diabetes mellitus, immune reactions in the pancreas cause the secretion of pro-inflammatory cytokines, such as interferon-Y. IFN-Y binding to JAK induces the pathway to up-regulate the transcription of genes, including those for chemokines, BCL2 and interferon regulatory transcription factors, resulting in a pro-inflammatory environment, which affects the normal B islet cell number and function [46,47]. Additionally, STAT1 expression in B cells is required for the pathogenesis of autoimmune diabetes, as it is involved with B cell loss.

### Outcomes in COVID-19: a common risk

IL-6, one of the culprits involved in cytokine storm development in COVID patients, is an activator of the JAK-STAT pathway and is increased in the serum of diabetic and obese patients. A suggestion arises where manipulation of this pathway may offer a potential therapeutic approach particularly advantageous to obese and diabetic patients.

Interestingly, these two patient groups are at higher risk of poor outcomes in the setting of COVID-19 infection. Diabetes accounted for more than 20% of ICU admissions in a cohort study in China [48]. In Italy, two-thirds of those who died with SARS-Cov-2 had diabetes [49]. Obesity has been associated with a three-fold increased risk of severe COVID infection [50]. Visceral and liver fat is independently correlated with increased levels of IL-6, the key pro-inflammatory cytokine involved in the inflammatory storm [50,51].

Both diabetes and obesity potentiate cardiovascular risk factors, which are recognized to increase the risk of poor outcomes in the setting of COVID-19. In both patient groups, there is limited ‘ability to evoke an appropriate metabolic response upon immunologic challenge’ [52]. Given the dysregulated JAK-STAT pathway in these patient groups, it may be another potential mechanism that explains why these groups have increased risk of poor outcomes in infection.

## JAK-STAT Inhibitors; Are They Feasible?

### Use in the obese and diabetic

As mentioned before, the several JAK-STAT pathways implicated in the development of both obesity and diabetes have garnered the suggestion that inhibition of this pathway may provide an advantage for their use in these patient groups. Brosius *et al*. have outlined the potential of JAK inhibition in diabetic patients. JAK inhibition serves both an anti-inflammatory purpose as well as aid in renin angiotensin aldosterone system inhibition, uniquely treating diabetic kidney disease (DKD). Safety concerns arise when considering long-term therapy, especially with the potential of aggravating anemia, which often complicates those with DKD. Additionally, activation of some STAT pathway in diabetic patients can occur independent of JAK activation; intervention more distally in this cascade may be required [53].

In obesity, JAK inhibition has been shown to induce the browning of white adipose tissue as well as amelioration of obesity-related metabolic disorders *in vitro*. In murine models, thermogenic capacity was increased, whereas chronic inflammation of adipose was not ameliorated. JAK inhibition showed preserved insulin sensitivity, as well as a significant reduction in free fatty acids and serum triglycerides [54]. Interestingly, obesity appears to influence the effectiveness of therapies that target the immune response. JAK inhibitor use has shown a negative impact on low disease activity in obese patients with rheumatoid arthritis. Similarly, obese patients were found to have an inferior response to anti-TNF therapy in rheumatoid arthritis (RA), suggesting the role of obesity as an effect modifier and the need for weight loss in those with underlying immune-mediated inflammatory diseases who become resistant to immunological therapy [55].

### Use in the setting of COVID-19

JAK-STAT inhibitors in the treatment of COVID-19 patients offer a potential advantage in that they achieve their effects at already approved dosages, and some have minimal protein binding allowing for combination therapy with antiretrovirals [56]. However, evidence suggests that JAK-STAT inhibitors may cause reflares of latent viral infections, such as herpes zoster. Other concerns include lipid profile derangement, as well as decreases in neutrophils, lymphocytes, NK cells, platelets, and increases in transaminases and serum creatinine levels, all of which are mild and reversible [57]. However, their safety profile is still comparable and acceptable with other biologic drugs [58,59].

In the setting of COVID-19, Randomized Controlled Trials (RCTs) are many and still ongoing (i.e. tofacitinib [60], NCT04332042; baricitinib [61], NCT04321993, NCT04345289, NCT04320277, NCT04346147, NCT04340232; ruxolitinib, NCT04348695, NCT04331665, NCT04337359, NCT04338958, NCT04334044, NCT04348071).

Cantini *et al*. [62] assessed the safety profile of baricitinib combined with lopinavir–ritonavir in moderate COVID-19 pneumonia. Patients were treated for 2 weeks with baricitinib 4 mg/day added to lopinavir–ritonavir therapy. Treatment was well tolerated with no serious adverse events. Clinical characteristics and respiratory functions were much improved at weeks 1 and 2 compared to baseline. There were less ICU transfers, more discharges and more negative swabs on discharge in those treated with JAK inhibitors. No infections, cardiovascular or hematological adverse events occurred after 2 weeks of treatment.

### Concerns about side effects

Perhaps the most alarming concern regarding the use of JAK-inhibitors is due to their effect of inhibiting cytokines that are essential in the response against pathogens, especially interferons. There is still some argument regarding the use of immune modulators in the setting of COVID-19 acute infection. For example, some studies involving corticosteroids have yet to find any difference in clinical outcomes, whereas some report findings of negative outcomes [63]. Without the essential action of interferons on infection, patients may be put at unnecessary risk of bacterial superinfection, and prolonged and more severe disease course. Therefore, all medications that intend to target the various inflammatory immune pathways in the setting of infection need to be approached with considerable caution.

#### Reactivation of latent infection

Attention has been called to concerns of reactivations of latent viral infection in light of new COVID-19 patient data, where neutrophil and lymphocyte counts were found to be close to the threshold value in patients admitted to ICU and non-survivors [48,64–66].

Harigai *et al*. [67,68] have studied reactivations of infections in RA patients on baricitinib; monitoring for hepatitis B virus DNA in those with latent infection is recommended. The risk of tuberculosis (TB) was strongly associated with whether or not patients belonged to endemic TB areas. Screening is recommended in those with latent TB. The general risks involved with JAK-STAT inhibitors include an increased risk for herpes zoster common to all JAK inhibitors [68]. Fundamentally, there is no difference between JAK inhibitors and other disease-modifying anti-rheumatic drugs with regard to infections; except for herpes zoster [69], with the risk being highest with baricitinib.

#### Concerns for coagulopathy and cardiovascular incident

Given the setting of increased coagulopathy in COVID-19 patients, the Food and Drug Administration (FDA) has warned about an increased risk for thromboembolism with the use of JAK-STAT inhibitors [70]. The finding of the increased risk of pulmonary embolism and death in RA patients was seen with the use of tofacitinib 10 mg twice daily dose. Table 1 highlights information on the only three JAK inhibitors with FDA approval, along with recommendations [60,61,71,72].

**Table 1:** Drug data on suggested approved JAK-inhibitors^a^ with Food and Drug Administration approval

Drug	Dosages	Target	Safety	Recommendations
Tofacitinib	5 mg twice daily for rheumatoid arthritis (11 mg extended release available) 10 mg for UC	JAK1 JAK3	Risk of PE and death in rheumatoid arthritis patients on 10 mg. CYP34 substrate with CYP inhibition potential. No association with QT prolongation. Potential risk of gastrointestinal perforation in patients with intestinal diverticulum/diverticulitis.	Frequent monitoring for coagulopathy, and symptom of PE. If serum aminotransferase elevated more than 5x upper limit, cease medication or dose reduction. Dose adjustment necessary in case of hepatic impairment. Dose adjustment warranted if co-administered with CYP3A4 inhibitors, namely fluconazole and ketoconazole.
Baricitinib	2 mg twice daily for rheumatoid arthritis.	JAK1 JAK 2	Excreted mostly unchanged in urine. Not affected by CYP inhibitors or inducers. Limited incidence of decreased hemoglobin, increased LDL, HDL, creatinine, creatine phosphokinase. Renal secretion affected by probenecid. No association with QT prolongation. Potential risk of gastrointestinal perforation in patients with intestinal diverticulum/diverticulitis.	If serum aminotransferase elevated more than 5x upper limit, cease medication or dose reduction. No cross reactivity in risk for hepatic injury between other DMARDs or other JAKinibs; may consider combination therapy. Low potential for drug–drug interactions. Dose reduction to 2 mg for patients on OAT3 inhibitors Dose reduction if renal clearance 30–60 mL/min. Not recommend if creatinine clearance <30 mL/min. Interrupt dose if ALC less than 500. or ANC less than 100 or Hb less than 8. Monitor for increase in lipid parameters.
Upadacitinib	15 mg	JAK1	CYP3A4 substrate. At clinical concentrations, no known inhibition of drug metabolizing enzymes or transporters. Mild-moderate hepatic impairment, and renal impairment have no effects on pharmacodynamics. No QT prolongation.	No dose adjustment needed for renal or hepatic patients. Interrupt dose if ALC less than 500 or ANC less than 100 or Hb less than 8.

a ALC, absolute lymphocyte count; ANC, absolute neutrophil count; DMARDs, disease modifying anti-rheumatic drugs. ALL JAK inhibitors are contraindicated during pregnancy. Women of child bearing age should, should initiate contraceptives at least 1 week before use. Avoid their use in patients who are breastfeeding.

Scott *et al*. [73] outlined the risk of thromboembolic events with JAK inhibitor use. The thromboembolic risk was found to be 5 per 1000 patient-years with the use of baricitinib 4 mg daily. In another study, no association was found between baricitinib use and major adverse cardiovascular event, arterial thrombotic event and congestive heart failure [74].

### Tolerability

Studies on tolerability to JAK-inhibitors revealed that the most frequent treatment requiring adverse events were nausea, headache and dyspepsia. Nausea was most common in those treated with tofacitinib compared to placebo, and in all patients, it was only mild, occurred in the first hour of dosing, and resolved on day 1. There were no deaths, severe or serious adverse events, or discontinuations. Additionally, there were no events of neutropenia, leucopenia, infection or anemia. No subjects had liver enzyme levels >3 times upper limit, or creatinine clearance >1.3 times upper limit of normal. No clinically relevant changes were seen in white blood cell count or any other lab investigations. No significant changes were found regarding heart rate, respiratory rate, blood pressure and no significant ECG findings [75]. Trials involving baricitinib showed the most common adverse effects were URTI, headache, blood creatinine kinase increase, diarrhea, nausea, and bronchitis. Temporary interruptions of JAK inhibitor therapy in RA patients, with later re-initiation of therapy, were associated with an increase in disease symptoms [76].

### Conclusion

JAK-STAT plays a critical role in modulating the immune system [6] by providing necessary and vital chemokines that help fend off infection. However, given the setting of COVID-19, which is highlighted by a state of over inflammation, JAK-STAT pathway activation comes at a steep price; an aggravated immune response likely to the detriment of the patient. The polarizing decision to either dampen or enhance the immune response is still a matter of argument; however, given the overwhelming evidence that an erratic immune response is the likely culprit of increased morbidity and mortality in COVID-19 patients, the playing field has opened up for a vast variety of medications, which target the pathways of inflammation. It would appear that inhibiting the JAK-STAT pathway has some potential [23]. Its multifaceted effects, from lowering IL6, to inhibiting viral endocytosis, and dampening the effects of AGII, as well as a specific potential benefit for obese and diabetic patient groups, suggests that JAK-STAT inhibitors are definitely worthy of consideration. Concerns arise due to their side effects, which most notably include inhibiting interferon production. So far, baseline use of these medications has not been shown to increase the risk of poor outcomes in COVID-19.

Clinicians considering the use JAK-STAT inhibitors should do so with care and caution, with frequent monitoring for flares of latent infection and potential lab derangements. It is not recommended that JAK-STAT inhibitors be used in asymptomatic patients, as 80% of these patients are generally capable of clearing infection via endogenous antiviral mechanisms [17]. Furthermore, in patients whose disease progression is more than moderate, that is requiring ICU admission, dose reduction or avoidance completely is recommended given the fears of co-infection/reactivation of latent infection, as well as concerns for lymphocytopenia that may be associated with this disease stage.

Long-term studies are needed to further establish and cement the use of JAK-STAT inhibitors in the fight against COVID-19. The results of ongoing RCT involving JAK-STAT inhibitor use in COVID-19 patients will enrich and enhance our understanding of this drug class.

An exaggerated or an inhibited JAK-STAT response has been suggested in the great majority of immune-related pathologies. Finding a delicate balance will prove to be decisive.

## Acknowledgements

**Conflicts of interest**

There are no conflicts of interest.
